# *catena*-Poly[[dipyridine­mercury(II)]-μ-5-amino-2,4,6-triiodo­isophthalato]

**DOI:** 10.1107/S1600536808030468

**Published:** 2008-09-27

**Authors:** Yu Zhang, Jianying Zhao, Guodong Tang, Zhengjing Jiang

**Affiliations:** a Department of Chemistry, Huaiyin Teachers College, Huai’an 223300, Jiangsu, People’s Republic of China; b Key Laboratory for Soft Chemistry and Functional Materials of the Ministry of Education, Nanjing University of Science and Technology, 200 Xiaolingwei, Nanjing 210094, Jiangsu, People’s Republic of China

## Abstract

The reaction of mercury(II) chloride with 5-amino-2,4,6-triiodo­isophthalic acid in pyridine solution leads to the formation of the title compound, [Hg(C_8_H_2_I_3_NO_4_)(C_5_H_5_N)_2_]_*n*_. The structure contains a four-coordinate Hg^2+^ ion in a distorted tetra­hedral geometry, which lies on a crystallographic twofold axis. The Hg^2+^ ion is bonded to two N atoms from two pyridine ligands and two carboxylate O atoms from two 5-amino-2,4,6-triiodo­isophthalate anions. The two carboxyl­ate groups of individual 5-amino-2,4,6-triiodo­isophthalate anions act as a bridge to the Hg centers. This anion also resides on a twofold axis, which passes through the amino N and the *trans* standing I atoms. The Hg—O distance is 2.337 (6) and the Hg—N distance is 2.244 (8) Å.

## Related literature

For general background, see: Ziegler *et al.* (1997 ▶). For related structures, see: Bebout *et al.* (1998 ▶); Beck & Sheldrick (2008 ▶); Matković-Čalogović *et al.* (2002 ▶); Weil (2001 ▶).
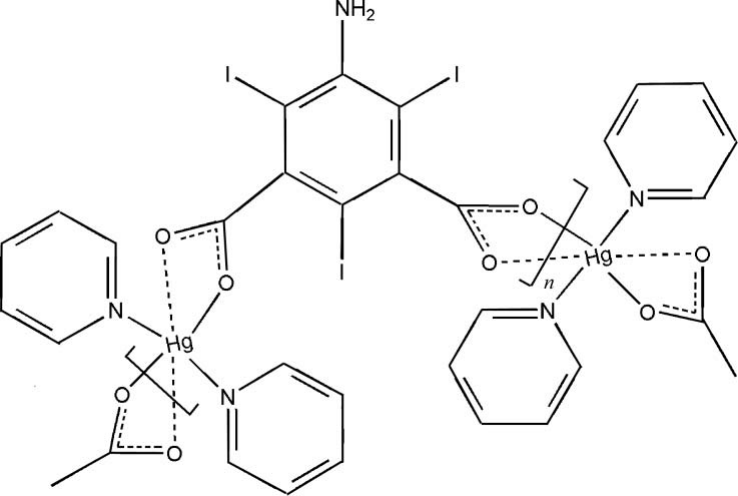


## Experimental

### 

#### Crystal data

[Hg(C_8_H_2_I_3_NO_4_)(C_5_H_5_N)_2_]
*M* *_r_* = 915.60
Tetragonal, 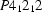

*a* = 11.9338 (3) Å
*c* = 16.0532 (10) Å
*V* = 2286.23 (16) Å^3^
*Z* = 4
Mo *K*α radiation
μ = 10.81 mm^−1^
*T* = 296 (2) K
0.20 × 0.15 × 0.15 mm

#### Data collection

Bruker SMART APEXII CCD diffractometer
Absorption correction: multi-scan (*SADABS*; Bruker, 2000 ▶) *T*_min_ = 0.16, *T*_max_ = 0.20
10399 measured reflections
2251 independent reflections
1661 reflections with *I* > 2σ(*I*)
*R*_int_ = 0.045

#### Refinement

*R*[*F* ^2^ > 2σ(*F* ^2^)] = 0.035
*wR*(*F* ^2^) = 0.067
*S* = 1.06
2251 reflections
134 parameters
57 restraints
H-atom parameters constrained
Δρ_max_ = 0.63 e Å^−3^
Δρ_min_ = −0.53 e Å^−3^
Absolute structure: Flack (1983 ▶), 897 Friedel pairs
Flack parameter: 0.010 (9)

### 

Data collection: *APEX2* (Bruker, 2004 ▶); cell refinement: *SAINT* (Bruker, 2004 ▶); data reduction: *SAINT*; program(s) used to solve structure: *SHELXS97* (Sheldrick, 2008 ▶); program(s) used to refine structure: *SHELXL97* (Sheldrick, 2008 ▶); molecular graphics: *SHELXTL* (Sheldrick, 2008 ▶); software used to prepare material for publication: *SHELXTL*.

## Supplementary Material

Crystal structure: contains datablocks I, global. DOI: 10.1107/S1600536808030468/fj2148sup1.cif

Structure factors: contains datablocks I. DOI: 10.1107/S1600536808030468/fj2148Isup2.hkl

Additional supplementary materials:  crystallographic information; 3D view; checkCIF report

## Figures and Tables

**Fig. 1.**
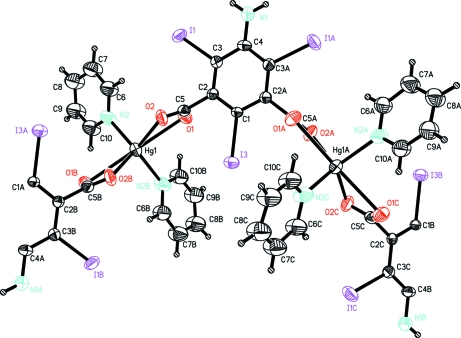
The molecular structure of title compound, with atom labels and 30% probability displacement ellipsoids. All H atoms have been omitted.

## supplementary crystallographic information

### Comment

5-amino-2, 4, 6-triiodoisophthalic acid is the precursor of the synthesis of a wide range of contrast agents with different amide-bound aliphatic side chains, which modulate their physical and physiological properties (Ziegler *et al.*, 1997). The crystal structure of this compound was reported very recently (Beck *et al.*, 2008), however, there is no information about the structural characterization of its metal complexes. In the present study, the synthesis, crystal structures of the *catena*-[bis(pyridine)- 5-Amino-2,4,6-triiodoisophthalic acid-O,*O* -mercury(II)] complex is reported.

The molecular structure of title complex comprises of two crystallographically independent chains along the *c*-axis. In the chains, Hg atom shows a distorted tetrahedron environment with [2 N + 2O] coordination, where two nitrogen atoms originate from pyridines and two oxygen atoms from two 5-amino-2,4,6-triiodoisophthalic acid ligands. The two CO_2_^-^ group of 5-Amino-2,4,6 -triiodoisophthalic acid ligand coordinated to Hg^2+^ and bridging Hg metal centers. The bond lengths of Hg—O in Table 1 indicate that the Hg (II) center is in a distorted. The bond lengths are and 2.337 (6)Å for Hg1—O2. There is a week interaction between Hg^2+^ and O1 with Hg1—O1 distance is 2.618 (6) Å The Hg—O bond lengths of this complex are close to the Hg—O bond lengths in the reported coordination polymers aqua-bromo(6-carboxypyridine-2-carboxylato –O, N, O') mercury(II) (2.425 (4)Å and 2.599 (4) Å) (Matković-Calogović *et al.*, 2002). The bond angles O1—Hg1—O2 existing in the octahedral are 53.1 (2)°, respectively, which also demonstrate the distorted octahedron in the Hg coordination center. The bond lengths of O1—C5 and O2—C5, are 1.245 (10)Å and 1.257 (10) Å, respectively, and the bond angle of O1—C1—O2 is 125.8 (8)°. Compared with the data when the ligand has not been coordinated (Beck *et al.*, 2008), the C—O bond lengths are lengthened and the O—C—O bond angles are slightly expanded when the carboxylate groups are coordinated to central cations. The C—I and C—N bond distances in the complex are very similar with the free ligand (Beck *et al.*, 2008). Two pyridine ligands coordinated to every Hg center with the Hg—N bond distances 2.444 (8) Å, which are fall in the range of the Hg—N bond lengths in the reported coordination compound mercury(II) Complexes of bis[(2-pyridyl)methyl]amine (in which, the Hg—N (pyridyl) bond lengths are range from 2.352 (4)Å to 2.557 (5) Å) (Bebout, *et al.*, 1998)and significantly longer than that in bis(pyridine-N)mercury(II) dichromate(VI)(in which, the average Hg—N distance is 2.101 Å) (Weil, 2001).

### Experimental

0.27 g (1 mmol) HgCl_2_ was dissolved in 10 mL ethanol, 0.54 g (1 mmol) 5-amino-2, 4, 6-triiodoisophthalic acid was dissolved in 10 mL pyridine. To mix two solutions gave slightly brown solution which was stirred at room temperature for 2 h, then filtered. The filtrate was stood for several days until the well formed colorless single crystals were obtained.

### Refinement

H atoms were positioned geometrically and refined with riding model, with *U*_iso_ = 1.5*U*_eq_ for methyl H atoms.

### Crystal data

**Table:**

[Hg(C_8_H_2_I_3_NO_4_)(C_5_H_5_N)_2_]	*D*_x_ = 2.660 Mg m^−^^3^
*M**_r_* = 915.60	Mo *K*α radiation, λ = 0.71073 Å
Tetragonal, *P*4_1_2_1_2	Cell parameters from 2411 reflections
Hall symbol: P 4abw 2nw	θ = 2.1–25.1°
*a* = 11.9338 (3) Å	µ = 10.81 mm^−^^1^
*c* = 16.0532 (10) Å	*T* = 296 K
*V* = 2286.23 (16) Å^3^	Pyramid, colorless
*Z* = 4	0.20 × 0.15 × 0.15 mm
*F*(000) = 1648	

### Data collection

**Table:**

Bruker SMART APEX CCD diffractometer	2251 independent reflections
Radiation source: fine-focus sealed tube	1661 reflections with *I* > 2σ(*I*)
graphite	*R*_int_ = 0.045
φ and ω scans	θ_max_ = 26.0°, θ_min_ = 2.1°
Absorption correction: multi-scan (SADABS; Bruker, 2000)	*h* = −11→14
*T*_min_ = 0.16, *T*_max_ = 0.20	*k* = −14→12
10399 measured reflections	*l* = −19→13

### Refinement

**Table:**

Refinement on *F*^2^	Secondary atom site location: difference Fourier map
Least-squares matrix: full	Hydrogen site location: inferred from neighbouring sites
*R*[*F*^2^ > 2σ(*F*^2^)] = 0.035	H-atom parameters constrained
*wR*(*F*^2^) = 0.067	*w* = 1/[σ^2^(*F*_o_^2^) + (0.0151*P*)^2^] where *P* = (*F*_o_^2^ + 2*F*_c_^2^)/3
*S* = 1.06	(Δ/σ)_max_ = 0.001
2251 reflections	Δρ_max_ = 0.63 e Å^−^^3^
134 parameters	Δρ_min_ = −0.53 e Å^−^^3^
57 restraints	Absolute structure: Flack (1983)
Primary atom site location: structure-invariant direct methods	Flack parameter: 0.010 (9)

### Special details

**Table:**

Geometry. All e.s.d.'s (except the e.s.d. in the dihedral angle between two l.s. planes) are estimated using the full covariance matrix. The cell e.s.d.'s are taken into account individually in the estimation of e.s.d.'s in distances, angles and torsion angles; correlations between e.s.d.'s in cell parameters are only used when they are defined by crystal symmetry. An approximate (isotropic) treatment of cell e.s.d.'s is used for estimating e.s.d.'s involving l.s. planes.
Refinement. Refinement of *F*^2^ against ALL reflections. The weighted *R*-factor *wR* and goodness of fit *S* are based on *F*^2^, conventional *R*-factors *R* are based on *F*, with *F* set to zero for negative *F*^2^. The threshold expression of *F*^2^ > σ(*F*^2^) is used only for calculating *R*-factors(gt) *etc*. and is not relevant to the choice of reflections for refinement. *R*-factors based on *F*^2^ are statistically about twice as large as those based on *F*, and *R*- factors based on ALL data will be even larger.

### Fractional atomic coordinates and isotropic or equivalent isotropic displacement parameters (Å^2^)

**Table:**

	*x*	*y*	*z*	*U*_iso_*/*U*_eq_	Occ. (<1)
C1	0.6137 (6)	0.3863 (6)	0.7500	0.036 (2)	
C2	0.6218 (7)	0.3118 (6)	0.6838 (5)	0.041 (2)	
C3	0.7046 (7)	0.2319 (7)	0.6843 (5)	0.046 (2)	
C4	0.7809 (7)	0.2191 (7)	0.7500	0.049 (3)	
C5	0.5357 (7)	0.3173 (8)	0.6152 (6)	0.051 (2)	
C6	0.4694 (10)	0.1635 (9)	0.4089 (8)	0.097 (2)	
H6	0.5308	0.1844	0.4410	0.116*	
C7	0.4795 (12)	0.0725 (11)	0.3565 (8)	0.112 (2)	
H7	0.5451	0.0305	0.3549	0.134*	
C8	0.3905 (11)	0.0459 (11)	0.3071 (9)	0.118 (2)	
H8	0.3965	−0.0087	0.2660	0.142*	
C9	0.2919 (11)	0.1019 (10)	0.3198 (8)	0.111 (2)	
H9	0.2264	0.0787	0.2937	0.133*	
C10	0.2919 (10)	0.1900 (10)	0.3703 (8)	0.098 (2)	
H10	0.2266	0.2322	0.3738	0.118*	
Hg1	0.37049 (3)	0.37049 (3)	0.5000	0.05760 (17)	
I1	0.72004 (6)	0.12434 (7)	0.58073 (4)	0.0764 (2)	
I3	0.48846 (5)	0.51154 (5)	0.7500	0.0606 (2)	
N1	0.8605 (6)	0.1395 (6)	0.7500	0.065 (3)	
H1A	0.9060	0.1340	0.7914	0.077*	0.50
H1B	0.8660	0.0940	0.7086	0.077*	0.50
N2	0.3772 (8)	0.2212 (7)	0.4151 (5)	0.0831 (19)	
O1	0.4543 (5)	0.2528 (6)	0.6227 (4)	0.0659 (18)	
O2	0.5496 (5)	0.3874 (6)	0.5577 (4)	0.0669 (18)	

### Atomic displacement parameters (Å^2^)

**Table:**

	*U*^11^	*U*^22^	*U*^33^	*U*^12^	*U*^13^	*U*^23^
C1	0.033 (4)	0.033 (4)	0.041 (6)	0.002 (5)	0.006 (4)	0.006 (4)
C2	0.041 (5)	0.043 (5)	0.039 (5)	0.002 (4)	0.011 (5)	0.010 (4)
C3	0.045 (5)	0.049 (5)	0.043 (5)	0.002 (4)	−0.002 (4)	0.000 (4)
C4	0.042 (4)	0.042 (4)	0.062 (8)	0.009 (6)	0.004 (5)	0.004 (5)
C5	0.039 (5)	0.062 (6)	0.051 (6)	0.008 (3)	−0.003 (4)	−0.008 (5)
C6	0.091 (5)	0.089 (5)	0.111 (5)	0.019 (4)	−0.038 (4)	−0.034 (4)
C7	0.104 (5)	0.099 (5)	0.132 (6)	0.027 (4)	−0.041 (4)	−0.043 (4)
C8	0.113 (5)	0.103 (5)	0.138 (6)	0.023 (4)	−0.046 (5)	−0.051 (4)
C9	0.103 (5)	0.096 (5)	0.133 (6)	0.015 (4)	−0.052 (5)	−0.045 (4)
C10	0.092 (5)	0.085 (5)	0.117 (5)	0.014 (4)	−0.046 (4)	−0.033 (4)
Hg1	0.0627 (2)	0.0627 (2)	0.0475 (3)	0.0051 (3)	−0.0068 (2)	0.0068 (2)
I1	0.0676 (4)	0.0958 (6)	0.0658 (5)	0.0210 (4)	0.0003 (4)	−0.0324 (4)
I3	0.0547 (3)	0.0547 (3)	0.0723 (6)	0.0119 (4)	0.0044 (3)	0.0044 (3)
N1	0.070 (4)	0.070 (4)	0.053 (6)	0.004 (7)	−0.004 (4)	−0.004 (4)
N2	0.081 (4)	0.072 (4)	0.097 (4)	0.013 (3)	−0.040 (4)	−0.021 (3)
O1	0.050 (4)	0.077 (5)	0.071 (4)	−0.001 (3)	−0.017 (3)	0.008 (3)
O2	0.068 (4)	0.088 (5)	0.044 (4)	0.001 (4)	−0.006 (3)	0.020 (4)

### Geometric parameters (Å, °)

**Table:**

C1—C2^i^	1.389 (9)	C8—C9	1.368 (15)
C1—I3	2.113 (9)	C8—H8	0.9300
C2—C3	1.373 (10)	C9—C10	1.327 (15)
C2—C5	1.508 (12)	C9—H9	0.9300
C3—C4	1.402 (10)	C10—N2	1.302 (12)
C3—I1	2.108 (8)	C10—H10	0.9300
C4—N1	1.344 (14)	Hg1—N2	2.244 (8)
C4—C3^i^	1.402 (10)	Hg1—N2^ii^	2.244 (8)
C5—O2	1.257 (10)	Hg1—O2^ii^	2.337 (6)
C5—O1	1.245 (10)	Hg1—O2	2.337 (6)
C5—Hg1	2.777 (9)	Hg1—O1^ii^	2.618 (6)
C6—N2	1.302 (12)	Hg1—O1	2.618 (6)
C6—C7	1.379 (15)	Hg1—C5^ii^	2.777 (9)
C6—H6	0.9300	N1—H1A	0.8600
C7—C8	1.363 (15)	N1—H1B	0.8600
C7—H7	0.9300		
			
C2^i^—C1—C2	119.7 (9)	N2^ii^—Hg1—O2^ii^	106.0 (3)
C2^i^—C1—I3	120.2 (5)	N2—Hg1—O2	106.0 (3)
C2—C1—I3	120.2 (5)	N2^ii^—Hg1—O2	118.8 (3)
C3—C2—C1	119.4 (8)	O2^ii^—Hg1—O2	89.9 (3)
C3—C2—C5	121.6 (8)	N2—Hg1—O1^ii^	82.3 (2)
C1—C2—C5	118.9 (7)	N2^ii^—Hg1—O1^ii^	91.0 (3)
C2—C3—C4	123.1 (8)	O2^ii^—Hg1—O1^ii^	53.1 (2)
C2—C3—I1	118.8 (6)	O2—Hg1—O1^ii^	139.1 (2)
C4—C3—I1	118.1 (6)	N2—Hg1—O1	91.0 (3)
N1—C4—C3^i^	122.4 (5)	N2^ii^—Hg1—O1	82.3 (2)
N1—C4—C3	122.4 (5)	O2^ii^—Hg1—O1	139.1 (2)
C3^i^—C4—C3	115.2 (10)	O2—Hg1—O1	53.1 (2)
O2—C5—O1	125.8 (8)	O1^ii^—Hg1—O1	167.5 (3)
O2—C5—C2	118.4 (8)	N2—Hg1—C5	101.4 (3)
O1—C5—C2	115.8 (8)	N2^ii^—Hg1—C5	99.6 (3)
O2—C5—Hg1	56.8 (4)	O2^ii^—Hg1—C5	114.3 (3)
O1—C5—Hg1	69.6 (5)	O2—Hg1—C5	26.7 (2)
C2—C5—Hg1	168.6 (6)	O1^ii^—Hg1—C5	165.8 (3)
N2—C6—C7	122.5 (12)	O1—Hg1—C5	26.5 (2)
N2—C6—H6	118.7	N2—Hg1—C5^ii^	99.6 (3)
C7—C6—H6	118.7	N2^ii^—Hg1—C5^ii^	101.4 (3)
C6—C7—C8	118.0 (13)	O2^ii^—Hg1—C5^ii^	26.7 (2)
C6—C7—H7	121.0	O2—Hg1—C5^ii^	114.3 (3)
C8—C7—H7	121.0	O1^ii^—Hg1—C5^ii^	26.5 (2)
C9—C8—C7	118.0 (12)	O1—Hg1—C5^ii^	165.8 (3)
C9—C8—H8	121.0	C5—Hg1—C5^ii^	140.2 (4)
C7—C8—H8	121.0	C4—N1—H1A	120.0
C10—C9—C8	118.5 (12)	C4—N1—H1B	120.0
C10—C9—H9	120.7	H1A—N1—H1B	120.0
C8—C9—H9	120.7	C6—N2—C10	117.8 (10)
N2—C10—C9	124.4 (12)	C6—N2—Hg1	119.8 (7)
N2—C10—H10	117.8	C10—N2—Hg1	122.4 (7)
C9—C10—H10	117.8	C5—O1—Hg1	83.9 (5)
N2—Hg1—N2^ii^	115.2 (5)	C5—O2—Hg1	96.5 (5)
N2—Hg1—O2^ii^	118.8 (3)		

Symmetry codes: (i) −*y*+1, −*x*+1, −*z*+3/2; (ii) *y*, *x*, −*z*+1.
